# Phenotypic and Functional Characterization of Long-Term Cryopreserved Human Adipose-derived Stem Cells

**DOI:** 10.1038/srep09596

**Published:** 2015-04-15

**Authors:** Kar Wey Yong, Belinda Pingguan-Murphy, Feng Xu, Wan Abu Bakar Wan Abas, Jane Ru Choi, Siti Zawiah Omar, Mat Adenan Noor Azmi, Kien Hui Chua, Wan Kamarul Zaman Wan Safwani

**Affiliations:** 1 Department of Biomedical Engineering, Faculty of Engineering, University of Malaya, Lembah Pantai, 50603 Kuala Lumpur, Malaysia; 2 Bioinspired Engineering and Biomechanics Center (BEBC), Xi'an Jiaotong University, Xi'an 710049, P.R. China; 3 The Key Library of Biomedical Information Engineering of Ministry of Education, School of Life Science and Technology, Xi'an Jiaotong University, Xi'an 710049, P.R. China; 4 Department of Obstetrics and Gynaecology, Faculty of Medicine, University of Malaya, Lembah Pantai, 50603 Kuala Lumpur, Malaysia; 5 Department of Physiology, Faculty of Medicine, Universiti Kebangsaan Malaysia, Jalan Yaacob Latiff, 56000 Kuala Lumpur, Malaysia

## Abstract

Cryopreservation represents an effective technique to maintain the functional properties of human adipose-derived stem cells (ASCs) and allows pooling of cells via long-term storage for clinical applications, *e.g.*, cell-based therapies. It is crucial to reduce freezing injury during the cryopreservation process by loading the ASCs with the optimum concentration of suitable cryoprotective agents (CPAs). In this study, human ASCs were preserved for 3 months in different combinations of CPAs, including 1) 0.25 M trehalose; 2) 5% dimethylsulfoxide (DMSO); 3) 10% DMSO; 4) 5% DMSO + 20% fetal bovine serum (FBS); 5) 10% DMSO + 20% FBS; 6) 10% DMSO + 90% FBS. Interestingly, even with a reduction of DMSO to 5% and without FBS, cryopreserved ASCs maintained high cell viability comparable with standard cryomedium (10% DMSO + 90% FBS), with normal cell phenotype and proliferation rate. Cryopreserved ASCs also maintained their differentiation capability (*e.g.*, to adipocytes, osteocytes and chondrocytes) and showed an enhanced expression level of stemness markers (*e.g.*, NANOG, OCT-4, SOX-2 and REX-1). Our findings suggest that 5% DMSO without FBS may be an ideal CPA for an efficient long-term cryopreservation of human ASCs. These results aid in establishing standardized xeno-free long-term cryopreservation of human ASCs for clinical applications.

Stem cells hold great potential for many biomedical applications, particularly cell-based therapies and regenerative medicine^1^,^2^ due to their capability of self-renewing and differentiating into multiple specific types of cells^3^,^4^. Among various stem cells, adipose-derived stem cells (ASCs) have attracted special attention due to their readily accessibility and the abundance of adipose tissue^5^,^6^,^7^. However, whilst intensive applications require a large number of cells, *in vitro* expansion of ASCs may not yield sufficient cell numbers in a short duration. Therefore, the cells should be capable of being preserved in the long-term with cell functionality maintained for off-the-shelf use^8^. Cryopreservation may be an ideal option for this, which is currently the only method to preserve ASCs with maintained functional properties and genetic characteristics in the long term^9^.

Various methods have been developed for cryopreservation of various stem cells including human ASCs, such as slow freezing and vitrification^10^,^11^,^12^,^13^,^14^,^15^. Vitrification only fits well with cryopreservation of human cells in small volumes such as oocytes^16^ but is ill suited to large volumes of ASCs^17^. Further, it might lead to potential contamination with pathogenic agents due to the direct exposure of cells to non-sterile liquid nitrogen^18^, and it involves the potential issue of cell loss due to inefficient cell collection. Thus far, the slow freezing method is the most preferable method of cell cryopreservation in research laboratories today, due to the low risk of contamination and it being an easier process^19^. However, slow freezing results in a high risk of freeze injury (*e.g.*, cell death) due to the formation of intra- and extracellular ice during the freezing process^20^,^21^,^22^. To address this issue, optimization of the use of cryoprotective agents (CPAs) is very important to avoid the formation of ice crystal by loading the ASCs with the optimum concentration of suitable CPAs. Among various CPAs, 10% DMSO and FBS is the most widely used for cryopreservation of large volumes of cells and tissues including ASCs^10^,^11^,^13^,^15^. However, DMSO is cytotoxic at temperature > 4 °C^23^. The clinical uses of frozen/thawed cells preserved with 10% DMSO have caused many adverse effects and toxic reactions such as respiratory depression and neurotoxicity^24^,^25^. Further, the use of FBS in cryopreservation media should be minimized due to its potential to trigger xenogeneic immune response or transmit pathogens to the recipient^23^,^26^. However, there is still an unmet need for an alternative cryomedium to replace DMSO and FBS completely.

To replace DMSO, polyvinylpyrrolidone (PVP) and methylcellulose have been used as CPAs^12^,^14^, however they are less efficient than DMSO in terms of maintaining ASC viability. Further, the assessment of cryopreserved ASCs has been performed mostly in a qualitative manner, *e.g.*, differentiation potential, which was only determined with a histochemical staining method^14^. However, the comparison of expression levels of differentiation and stemness markers among ASCs preserved in various CPAs has not been explored yet. Herein, we performed quantitative assessments to accurately compare the effects of various combinations of CPA (DMSO, trehalose and FBS) on human ASCs in term of cell phenotype, proliferation potential, differentiation potential, stemness and viability. Based on the comparison, we determined the ideal CPA to preserve human ASCs effectively for clinical applications. The findings from this study would impact the establishment of standardized cryopreservation protocol of human ASCs for future clinical applications.

## Methods

### ASCs isolation and culture

This study was approved by the Medical Ethics Committee of University Malaya Medical Centre (UMMC) and was carried out in accordance with the approved guidelines. With prior informed written consent, adipose tissues samples were obtained from 6 different female donors (25–35 years old) undergoing caesarean section. All experimental protocols were approved by the Medical Ethics Committee of UMMC (reference no. 996.46). Adipose tissues were washed with phosphate buffer saline (PBS) (Sigma Aldrich, St. Louis, USA) and minced into small pieces. Tissue digestion was performed using 0.3% collagenase type I enzyme (Worthington, New Jersey, USA) solution at 37°C with agitation. The digested adipose tissues were centrifuged to obtain the pellets. Finally, the pellets were resuspended with culture medium composed of Dulbecco's Modified Eagle's medium (DMEM)/Ham F-12, 10% FBS, 1% antibiotic/antimycotic, 1% glutamax (Gibco, New York, USA) and 1% vitamin C (Sigma), and seeded in 25 cm^2^ cell culture flask. Cells were cultured under 5% CO_2_. Cells were expanded until passage 3 and some of them are cryopreserved at the end of passage 2. Cells at passage 3 were required for all assays.

### Cryopreservation

About 1 × 10^6^ cells at passage 2 were detached from the flask and loaded into cryovials with cryomedium containing CPAs in DMEM/Ham F-12. The CPAs used are: 1) 0.25 M trehalose; 2) 5% DMSO; 3) 10% DMSO; 4) 5% DMSO + 20% FBS; 5) 10% DMSO + 20% FBS and 6) 10% DMSO + 90% FBS. All cryovials were kept at −80°C overnight and transferred to liquid nitrogen (−196°C) the next day. After 3 months of cryopreservation, the cells were thawed rapidly at 37°C, sub-cultured to passage 3 and used for evaluation of cell phenotype, viability and functional properties.

### Cell phenotyping

Cell phenotyping was conducted through microscopic examination and immunophenotyping. Microscopic examination was conducted to determine the morphology of cells and their adherent properties, whereas immunophenotyping was used to determine the level of surface markers expressed by ASCs. To perform immunophenotyping, cells were incubated with antibodies tagged with fluorochrome as follows: CD 90-FITC, CD 73-PE, CD 105-FITC, CD 44-FITC, HLA ABC-FITC, CD 14-PE, CD19-PE, CD 34-FITC, CD 45-FITC and HLA DPDQDR-FITC, each for 30 minutes on ice. All antibodies used were supplied by Becton Dickinson, San Jose, USA. Then, the cells were washed and resuspended with sheath fluid (Becton Dickinson). Finally, data were acquired using a flow cytometry system (BD FACSCanto II, Becton Dickinson).

### Cell proliferation assay

ASCs were seeded into the 24 well plate with cell density of 2 × 10^4^ cells/cm^2^ and incubated overnight for cell attachment. Resazurin assays were conducted after 24 hours (day 1), and on days 3, 7, 10 and 14. The absorbance of resazurin at 570 nm and 595 nm was measured using a microplate reader (Fluostar Optima, BMG Labtech, Germany). Finally, the cell numbers on each day of culture were determined.

### Cell differentiation potential assays

For inducing adipogenesis, ASCs were cultured in adipogenic induction medium composed of complete culture medium, 200 μM indomethacin (Sigma), 0.5 μM isobutyl-1-methylxanthine (Sigma), 1 μM dexamethasone (Sigma) and insulin (Becton Dickinson) for 21 days. After 21 days, differentiation of ASCs into adipogenic-like cells was indicated by the appearance of lipid droplets stained by Oil Red O (Sigma). Further, the adipogenic potential of ASCs before and after cryopreservation was confirmed with determination of gene expression levels of adipogenic markers using Taqman probe-based Real-Time Polymerase Chain Reaction (PCR). Peroxisome proliferator-activated receptor-γ (PPAR-γ) (Hs01115513_m1), fatty acid binding protein (FABP4) (Hs01086177_m1) and Lipoprotein lipase (LPL) (Hs00173425_m1) serve as markers for adipogenic differentiation.

To induce osteogenic differentiation, ASCs were cultured in osteogenic induction medium composed of complete culture medium, 100 nM dexamethasone (Sigma), 10 mM b-glycerophosphate (Sigma) and 0.5 mM ascorbic acid-2-phosphate (Sigma) for 21 days. After 21 days, differentiation of ASCs into osteogenic-like cells is indicated by calcium deposition stained using Alizarin Red (Sigma). Further, osteogenic potential of ASCs before and after cryopreservation was confirmed with determination of gene expression levels of osteogenic markers. Alkaline phosphatase (ALP) (Hs01029144_m1), Osteocalcin (OSC) (Hs015878914_m1) and Runt-related transcription factor 2 (Runx2) (Hs00231692_m1) serve as markers for osteogenic differentiation.

To induce chondrogenesis, ASCs in pellet form were cultured in chondrogenic induction medium composed of complete culture medium with 1% ITS (Becton Dickinson), 50 μg/ml ascorbate-2-phosphate (Sigma), 100 nM dexamethasone (Sigma), 40 μg/ml L-proline (Sigma), 10 ng/ml TGF-β1 (Peprotech, New Jersey, USA) and 50 ng/ml IGF-1 (Peprotech) for 21 days. After 21 days, the pellets were fixed in 4% neutral buffered formalin and then processed according to the standard histological procedures to produce tissue sections. Finally, the tissue sections were stained with Alcian Blue (Sigma). Differentiation of ASCs into chondrogenic-like cells is indicated by the presence of proteoglycan. Further, the chondrogenic potential of ASCs before and after cryopreservation was confirmed by determining the gene expression levels of chondrogenic markers. Aggrecan (ACAN) (Hs00153936_m1), Collagen type II (COL2A) (Hs00264051_m1) and Sry-related HMG box-9 (SOX9) (Hs00165814_m1) served as markers for chondrogenic differentiation.

### Cell viability assay

Post-thaw cell viability of ASCs was determined with the trypan blue exclusion method. The total number of dead cells and live cells was counted under a light microscope. Cell viability of ASCs preserved in various CPAs was determined.

### RNA extraction, cDNA synthesis and quantitative Real-Time polymerase chain reaction

RNA extraction was conducted using TRI reagent (Ambion, Austin, USA), followed by phase separation with chloroform (Fisher Scientific) and RNA precipitation with isopropanol (Sigma). The high capacity RNA-to-cDNA kit (Applied Biosystems, Foster City, USA) was used to synthesize cDNAs. Real-Time PCR was performed using *TaqMan* gene expression assays (Applied Biosystems) and Real-Time PCR machine (StepOnePlus, Applied Biosystems). The genes include differentiation markers (as mentioned earlier), stemness markers such as OCT-4 (Hs04260367_g1), REX-1 (Hs01938187_s1), SOX-2 (Hs01053049_s1) and NANOG (Hs01060663_m1). Housekeeping gene used for normalization was GAPDH (Hs99999905_m1). The gene expression level of the control group (fresh ASCs or ASCs before differentiation) was normalized to 1. All the results were expressed as fold changes in gene expression relative to the control.

### Statistical analysis

Statistical analysis was performed using One-Way ANOVA with tukey post hoc test to compare data among cryopreserved and fresh ASC groups. Data before and after the differentiation induction in the gene expression study were compared using a paired *t*-test. Each datum was expressed as mean ± standard error of mean (SEM). Statistical significance was accepted at *p*<0.05. All data analysis were performed using SPSS 17.0 software.

## Results and Discussions

### Effects of cryopreservation on ASC phenotype

**Figure 1:**
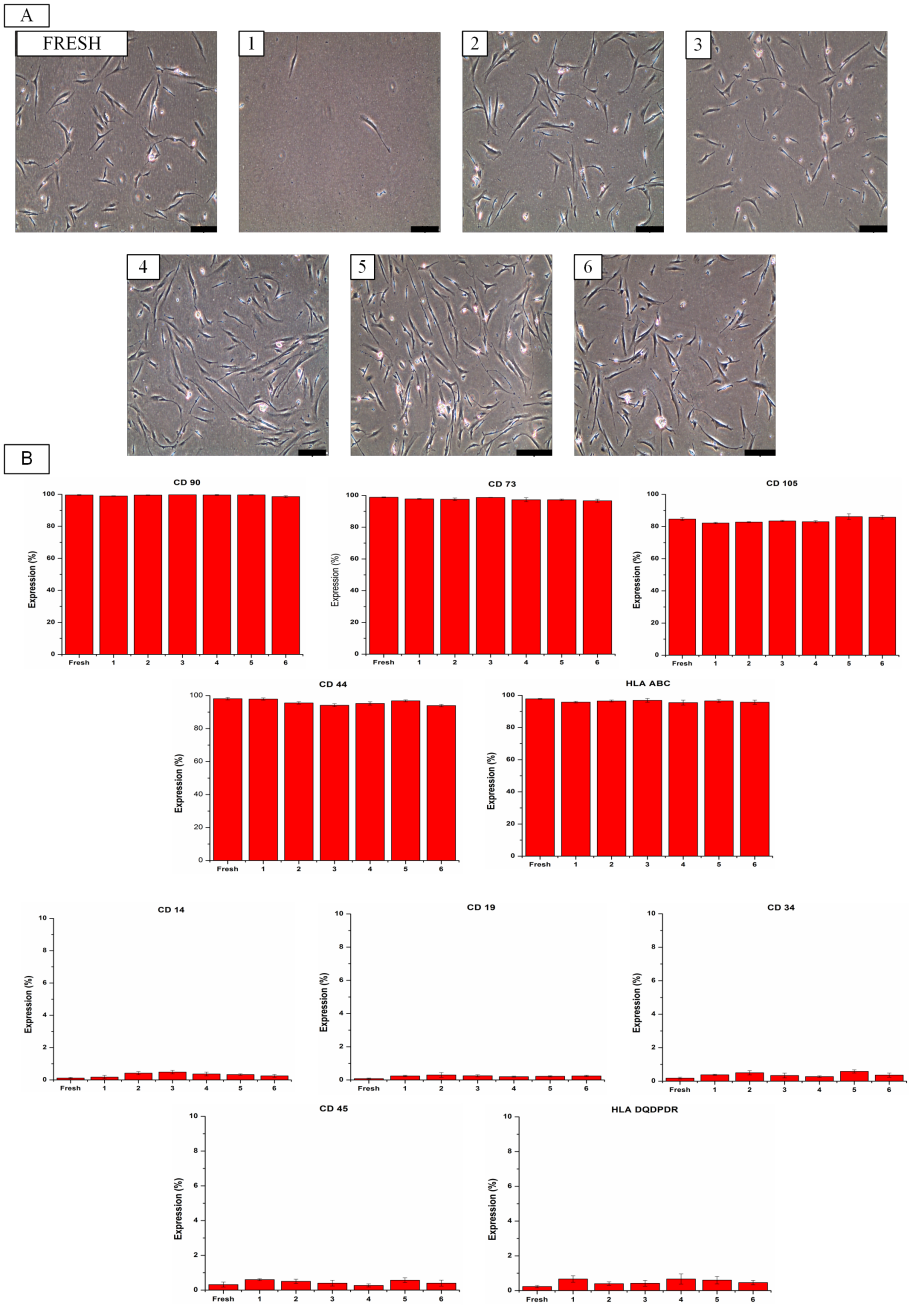
Cryopreservation maintained the phenotype of ASCs. (A) The fibroblast-like morphology of fresh and cryopreserved ASCs (magnification 100×). Scale bars: 100 µm. (B) Fresh and cryopreserved ASCs highly expressed positive markers (CD 90, CD 73, CD 105, CD 44 and HLA ABC) while lacked of negative markers (CD 14, CD 19, CD 34, CD 45 and HLA DRDPDQ). Cryoprotective agent: 1) 0.25 M trehalose; 2) 5% dimethylsulfoxide (DMSO); 3) 10% DMSO; 4) 5% DMSO + 20% fetal bovine serum (FBS); 5) 10% DMSO + 20% FBS; 6) 10% DMSO + 90% FBS.

In the present study, human ASCs were cryopreserved by a slow freezing method with various CPAs, including trehalose, DMSO and FBS. After 3 months of cryopreservation (long-term cryopreservation)^27^,^28^, cryopreserved ASCs were thawed and sub-cultured. To determine the effect of cryopreservation on ASC phenotype, we undertook microscopic examination and flow cytometry analysis. Through microscopic examination, we observed that cryopreserved and fresh ASCs (non-cryopreserved ASCs at passage 3) presented adherent and fibroblast-like shapes (Fig. 1A). Flow cytometry analysis showed that fresh and cryopreserved ASCs are positive for CD90, HLA ABC, CD44, CD105 and CD73 while negative for CD14, CD19, CD34, CD45 and HLA DRDPDQ (Fig. 1B). These results indicate that cryopreserved ASCs have retained fibroblast-like shapes and expressed similar pattern of cell surface markers as fresh ASCs, which is in accordance with the results reported by Gonda *et al.*^11^ and Liu *et al.*^10^. According to Dominici *et al.*^29^, ASCs should possess the criteria of MSCs, which are adherent cells with fibroblast-like shape^30^,^31^ and express the mesenchymal-associated markers (CD90, CD105 and CD73) while lack of hematopoietic-associated markers (CD14, CD19, CD34, CD45 and HLA DRDPDQ)^32^. Taken together, our findings suggest that phenotypes of ASCs were not affected by the cryopreservation process (freezing and thawing) and CPAs.

### Effects of cryopreservation on ASC viability and proliferation

**Figure 2:**
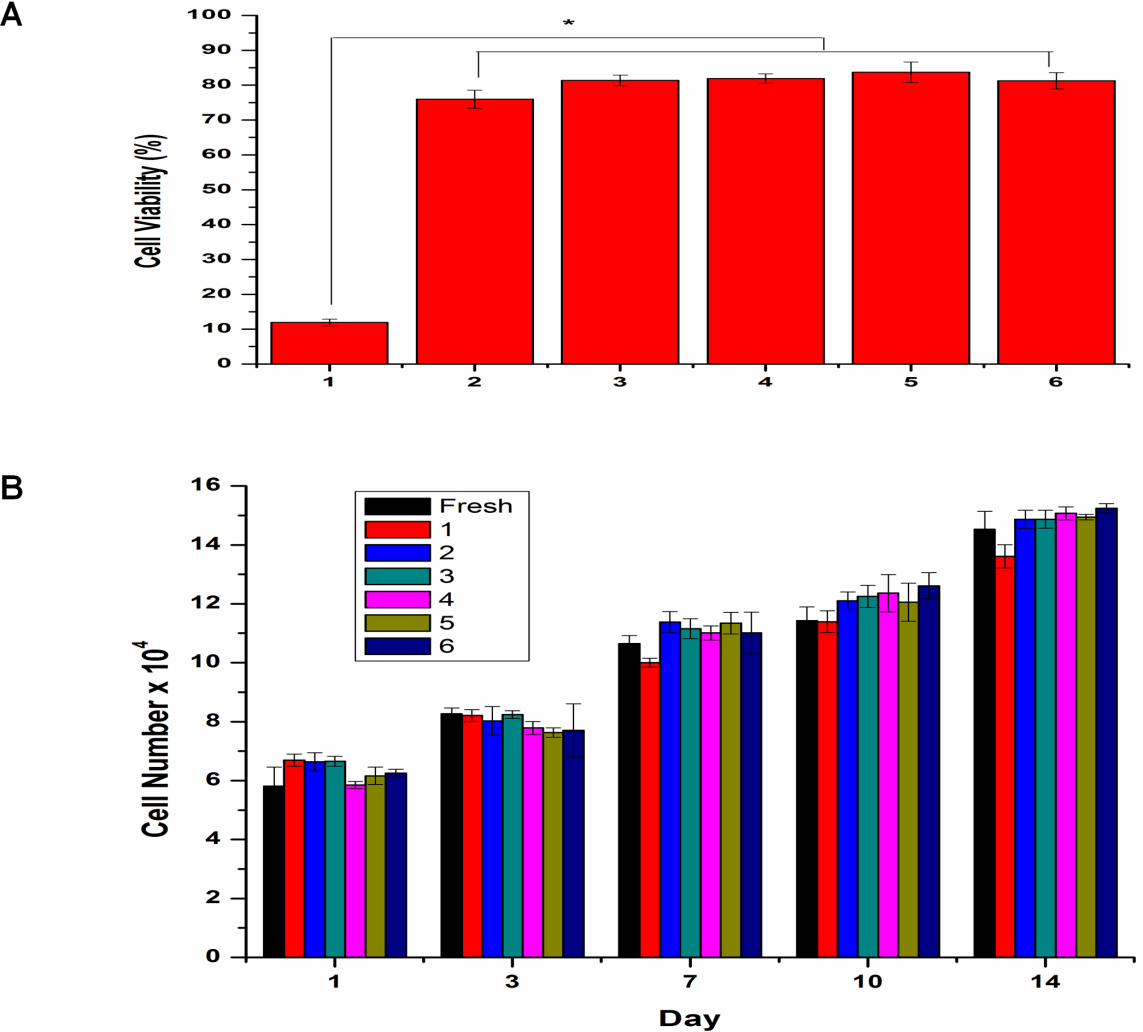
Effect of cryopreservation on ASCs viability and proliferation. (A) Cell viability of ASCs preserved in 5% dimethylsulfoxide (DMSO) is similar to those preserved in standard cryomedium (10% DMSO + 90% fetal bovine serum (FBS)), while significantly higher than those preserved in 0.25 M trehalose (*p<0.05 relative to 0.25 M trehalose). (B) Fresh and cryopreserved ASCs displayed similar cell number cultured from day 1 to day 14, indicating proliferative potential of ASCs were maintained after cryopreservation. Cryoprotective agent: 1) 0.25 M trehalose; 2) 5% DMSO; 3) 10% DMSO; 4) 5% DMSO + 20% FBS; 5) 10% DMSO + 20% FBS; 6) 10% DMSO + 90% FBS.

Besides maintaining their phenotype, ASCs should have the ability to survive long-term storage and maintain their functional properties if they are to be banked and used for clinical applications. Reductions in cell viability and functional capacity may have implications for the therapeutic application of ASCs^8^. Therefore, CPAs are essential to maintain the cell viability and functional properties when the cells are stored at -196 °C^33^,^34^. In this study, various well-known CPAs and combinations of CPAs were tested, including one intracellular compound (DMSO) that prevents ice crystal formation inside the cells^35^, and two extracellular agents (FBS and trehalose) that stabilize the cell membranes and adjust the osmotic pressure^36^. Interestingly, even with a reduction of DMSO CPA to 5% and without FBS, viability assays indicated cryopreserved ASCs have maintained a high cell viability comparable to those preserved in standard cryomedium (10% + 90% FBS)^37^,^38^. Meanwhile, ASCs preserved in 0.25 M trehalose showed the lowest cell viability *(p*<0.05) (Fig. 2A).

Trehalose led to a low viability of ASCs, possibly because trehalose cannot penetrate the cell membrane to prevent ice crystal formation within the cells, thus causing the rupture of cell membranes^33^,^39^. Eventually, such cells die due to dehydration and physical damage caused by intracellular ice. The higher cell viability from ASCs preserved in DMSO (compared to trehalose), indicates the relatively high efficiency of DMSO in maintaining the survival rate of ASCs throughout the freezing and thawing process. Due to the ability of penetrating cell membrane, DMSO protects the cell from rupture by removing the water within the cell to prevent intracellular ice crystals formation^35^. Janz *et al.*^40^ also showed that DMSO is better than trehalose in maintaining amniotic fluid-derived stem cells viability. Therefore, CPA containing trehalose alone cannot be used to replace DMSO in cryopreservation of ASCs. This result also indicates that exclusion of FBS in cryomedium does not cause any harmful effect on ASCs. Given that the CPA which contained 5% DMSO alone is less cytotoxic, and avoids the FBS which can activate xenogeneic immune reponses^26^, it raises its potential as a CPA to store cells for clinical applications. We also determined the cellular proliferation and found that cryopreserved and fresh ASCs displayed a similar cell number when cultured from day 1 till day 14 (Fig. 2B), suggesting that cryopreserved ASCs have a similar proliferation rate as fresh ASCs. This result showed that ASCs are capable of maintaining their proliferative potential after cryopreservation, which is supported by Gonda *et al.*^11^ and De Rosa *et al.*^13^.

### Effects of cryopreservation on ASC adipogenic potential

**Figure 3:**
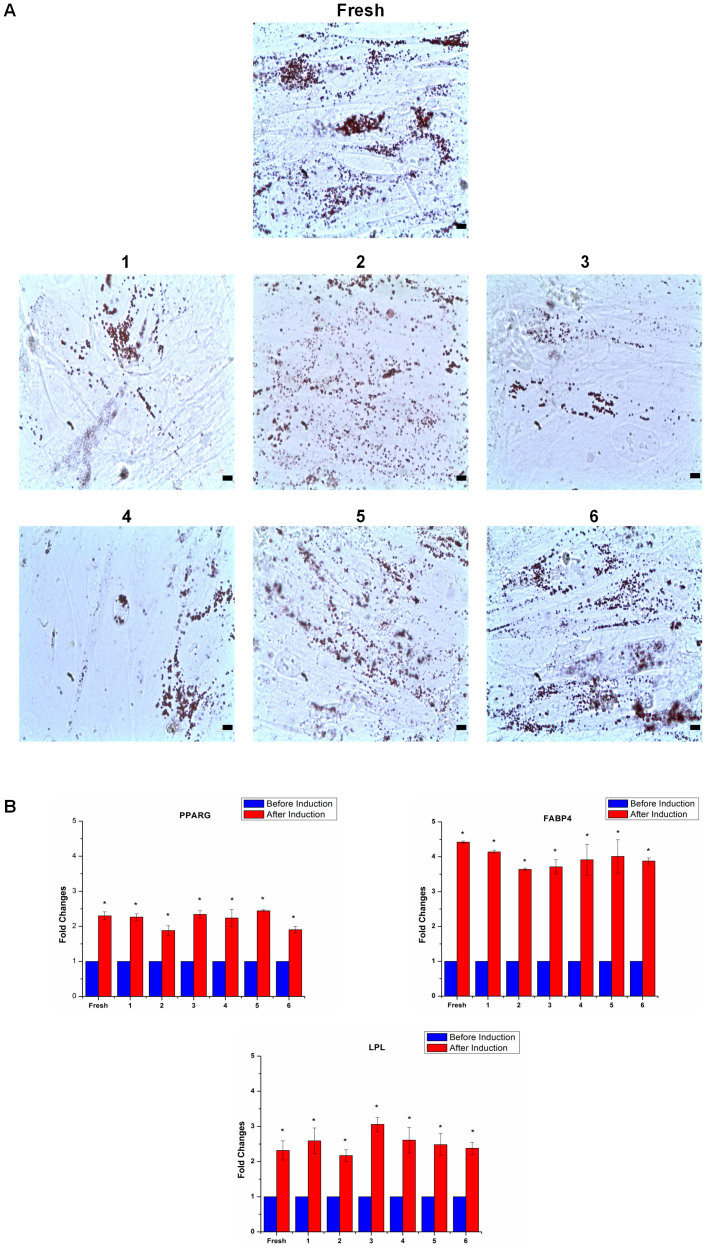
Cryopreservation maintained ASCs adipogenesis. (A) Adipogenesis assessed by Oil Red O staining (magnification 400×) (scale bars: 100 µm.). (B) Similar adipogenic gene (PPAR-γ, FABP4 and LPL) expression level in cryopreserved ASCs compared to fresh ASCs (*p<0.05 relative to before induction). Cryoprotective agent: 1) 0.25 M trehalose; 2) 5% dimethylsulfoxide (DMSO); 3) 10% DMSO; 4) 5% DMSO + 20% fetal bovine serum (FBS); 5) 10% DMSO + 20% FBS; 6) 10% DMSO + 90% FBS.

To assess adipogenic potential of ASCs after cryopreservation, we did Oil red O staining and adipogenic gene expression analysis. Upon adipogenesis, fresh and cryopreserved ASCs displayed formation of lipid droplets which were positively stained by Oil red O (Fig. 3A), which is supported by Gonda *et al.*^11^ and Thirumala *et al*.^14^ It has been reported that cryopreservation may decrease adipogenic and osteogenic potential of ASCs by downregulating the expression of adipogenic and osteogenic genes^15^. To provide an insight into molecular changes that may occur following cell freezing, we have checked the effect of cryopreservation on gene expression of differentiation capacity in ASCs. In this study, we have employed quantitative Real-Time PCR method to determine and compare the expression level of differentiation markers in fresh and cryopreserved ASCs. Both the fresh and cryopreserved ASCs showed significantly increased *(p*<0.05) expression level of adipogenic markers, including PPAR-γ, FBP4 and LPL after adipogenic differentiation induction (Fig. 3B). These results indicate that cryopreservation maintained ASCs capability to undergo adipogenesis. Similar expression level of adipogenic markers among fresh and ASCs preserved in various CPAs after adipogenic induction was observed, indicating that they possess similar adipogenic potential.

### Effects of cryopreservation on ASC osteogenic capacity

**Figure 4:**
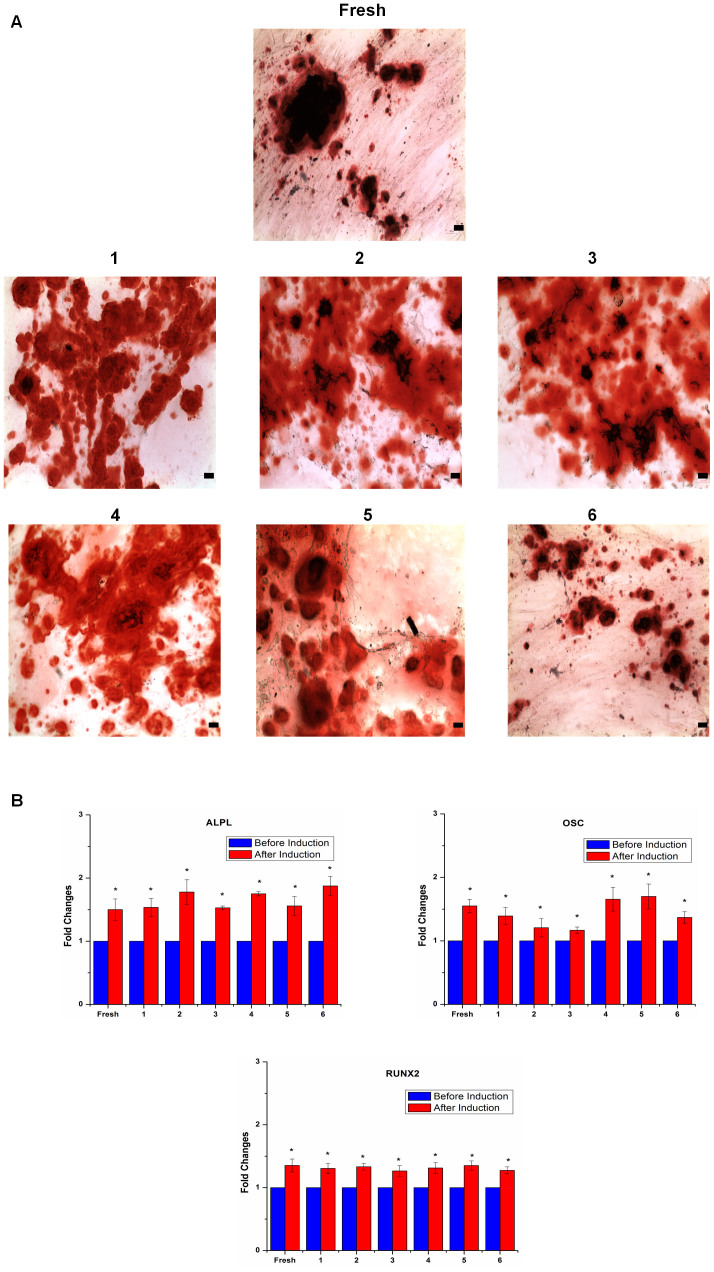
Cryopreservation maintained ASCs osteogenesis. (A) Osteogenesis indicated by Alizarin Red staining (magnification 100x) (scale bars: 100 µm). (B) Cryopreserved ASCs expressed similar level of osteogenic markers (ALPL, OSC and RUNX2) compared to fresh ASCs (*p<0.05 relative to before induction). Cryoprotective agent: 1) 0.25 M trehalose; 2) 5% dimethylsulfoxide (DMSO); 3) 10% DMSO; 4) 5% DMSO + 20% fetal bovine serum (FBS); 5) 10% DMSO + 20% FBS; 6) 10% DMSO + 90% FBS.

To determine osteogenic differentiation potential of cryopreserved ASCs, we conducted Alizarin Red staining and osteogenic gene expression analysis. Upon osteogenesis, calcium deposits were stained by Alizarin Red. We observed many dark red regions, indicating the abundant of calcium deposits in fresh ASCs and cryopreserved ASCs upon osteogenic differentiation induction (Fig. 4A). Similar results have been reported in literature^10^,^11^,^14^. Through Real-Time PCR, we observed that both fresh and cryopreserved ASCs have showed significantly increased *(p*<0.05) expression level of osteogenic markers, ALPL, OSC and RUNX2 after osteogenic induction (Fig. 4B). These results indicate that the osteogenic potential of ASCs was maintained after cryopreservation. There is no significant difference in expression level of osteogenic markers among fresh and ASCs preserved in various CPAs after osteogenic induction, suggesting that they have a similar osteogenic capacity.

### Effects of cryopreservation on ASC chondrogenic potential

**Figure 5:**
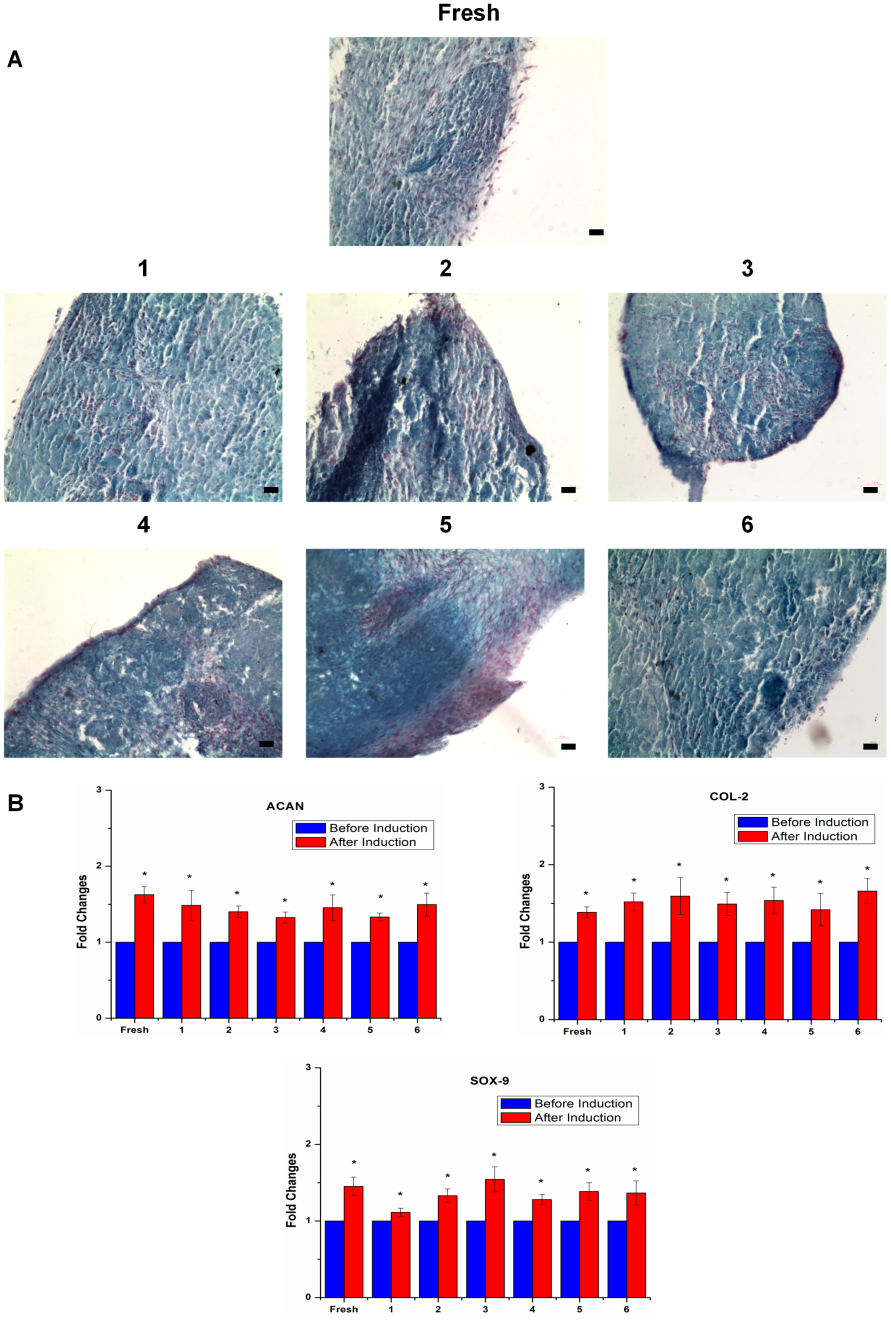
Chondrogenesis of ASCs were maintained after cryopreservation. (A) Chondrogenesis assessed by Alcian Blue staining (magnification 100×) (scale bars: 100 µm). (B) Cryopreserved and fresh ASCs expressed similar level of chondrogenic markers (ACAN, COL-2 and SOX-9) (*p<0.05 relative to before induction). Cryoprotective agent: 1) 0.25 M trehalose; 2) 5% dimethylsulfoxide (DMSO); 3) 10% DMSO; 4) 5% DMSO + 20% fetal bovine serum (FBS); 5) 10% DMSO + 20% FBS; 6) 10% DMSO + 90% FBS.

To evaluate the chondrogenic potential of ASCs after cryopreservation, we undertook Alcian blue staining and chondrogenic gene expression analysis. We observed that histological sections from both fresh and cryopreserved ASCs pellets cultured in chondrogenic induction media showed the formation of proteoglycan positively stained by Alcian Blue (Fig. 5A), which is in accordance with the findings reported by Gonda *et al*.^11^ The fresh and cryopreserved ASCs showed significantly increased *(p*<0.05) expression level of chondrogenic markers ACAN, COL-2 and SOX-9 after chondrogenikc induction (Fig. 5B). These results indicate that cryopreservation maintained ASC chondrogenic differentiation capacity. Similar expression level of chondrogenic markers among fresh and ASCs preserved in various CPAs after chondrogenic induction was observed, indicating that they possess similar chondrogenic potential.

Furthermore, we found that the increase in levels of the lineage markers in fresh and cryopreserved ASCs following adipogenic, osteogenic and chondrogenic (tri-lineage differentiation) induction in two-dimensional (2D) culture system are quite modest. Our result clearly showed that tri-lineage markers in ASCs are increased only within 1.5- and 4.5-fold upon tri-lineage differentiation induction, which were supported by Choi *et al.*^41^ and Bosnakovski *et al*.^42^ This is due to the fact that tri-lineage differentiation of MSCs is not only induced by the specific growth factors given, but also by the culture environment (*e.g.*, three-dimensional (3D) culture system) and the matrix (*e.g.*, collagen) the cells resided^43^,^44^. For instance, high levels of differentiation markers (increase more than 10-fold) following adipogenic, osteogenic and chondrogenic induction, were observed in MSCs encapsulated in 3D collagen hydrogels^42^,^44^. In future, we will extend our study in 3D microenvironment.

Overall, the results show that cryopreserved ASCs maintain their capabilities of differentiating into adipocytes, osteocytes and chondrocytes, which met the minimal criteria of characterizing the human MSCs as suggested by Dominici *et al*^29^. Taken together, cryopreserved ASCs, particularly ASCs preserved in 5% DMSO alone, can be potentially used in various clinical applications such as bone and cartilage regeneration due to their ability to maintain normal proliferation and differentiation potential.

### Effects of cryopreservation on ASC stemness

**Figure 6:**
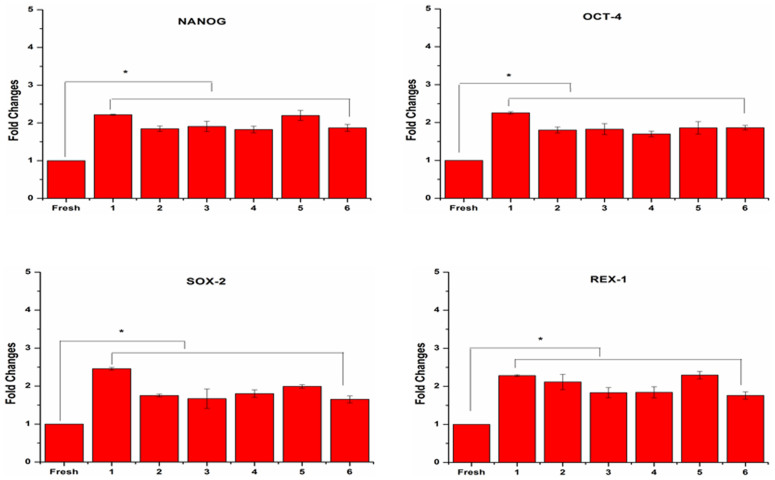
ASCs stemness were maintained after cryopreservation. Significant higher expression level of stemness markers (NANOG, OCT-4, SOX-2 and REX-1) were observed in cryopreserved ASCs (*p<0.05 relative to fresh ASCs). Cryoprotective agent: 1) 0.25 M trehalose; 2) 5% dimethylsulfoxide (DMSO); 3) 10% DMSO; 4) 5% DMSO + 20% fetal bovine serum (FBS); 5) 10% DMSO + 20% FBS; 6) 10% DMSO + 90% FBS.

Generally, stemness, a typical characteristic of MSCs, plays an important role in regulating self-renewal and differentiation activity^45^. Yoon *et al.*^46^ reported that knockdown of SOX-2 significantly inhibited cell proliferation and multipotency of MSCs. It has been reported that OCT-4 knockdown also significantly lowered the growth rates^47^ and multipotency of MSCs^48^. However, stemness markers analysis on cryopreserved ASCs has not been conducted yet. To determine the stemness markers expression in cryopreserved ASCs, we performed quantitative Real-Time PCR assays. We found that cryopreserved ASCs showed significantly higher (*p*<0.05) expression level in stemness genes including OCT-4, REX-1, SOX-2 and NANOG compared to fresh ASCs (Fig. 6), indicating their greater ability to maintain their stemness properties. However, the enhanced expression of stemness markers in cryopreserved human ASCs is yet to be explored, which requires further investigation. Fan *et al.*^49^ have reported that overexpression of SOX-2 in bone marrow-derived MSCs has resulted in higher proliferation and differentiation in term of adipogenesis and osteogenesis. OCT-4 overexpressed MSCs also have shown increased proliferation rate^50^. However, in this study, although cryopreserved ASCs showed increased expression level of stemness markers, they still displayed normal proliferation and differentiation potential. Choi *et al.*^41^ and Pierantozzi *et al.*^51^ have showed that increased proliferative and differentiation potential of MSCs is not directly associated with enhanced stemness markers expression. In fact, it has been reported that proliferation and differentiation activity of MSCs is mainly affected by other factors such as oxygen tension level^41^ and culture environment (two-dimensional (2D) or three-dimensional (3D))^44^. In short, cryopreserved ASCs maintained their stemness, including ASCs preserved in xeno-free cryomedium consists of 5% DMSO.

## Conclusions

In summary, long-term cryopreservation maintained cell phenotype, proliferation, differentiation and stemness of ASCs. Our findings suggest 5% DMSO without FBS may be an ideal CPA for long-term preservation of ASCs as it is less cytotoxic and leads to high rate of cell viability compared to standard cryomedium (10% + 90% FBS). Further, ASCs preserved in 5% DMSO without FBS were able to maintain their cell phenotype and functional properties, indicating their potential to be used for clinical therapies. Further investigation is needed to evaluate the biosafety of human ASCs preserved in 5% DMSO prior to clinical applications.

## Author Contributions

K.W.Y. and W.K.Z.W.S. designed the experiments. K.W.Y. performed the experiments and analysed the data. W.A.W.A. and B.PM. contributed instruments, reagents and materials. S.Z.O. and M.A.N.A. conducted surgeries to provide us adipose tissue samples. K.W.Y. wrote the manuscript while F.X., J.R.C., K.H.C. and W.K.Z.W.S. revised the manuscript. All authors reviewed the manuscript.
